# The Lectures of Sir Astley Cooper, Bart.

**Published:** 1826-04

**Authors:** 


### *The Lectures of Sir Astley Cooper, Bart. with additional Notes and Cases.* By Frederick Tyrrell, Esq.

[Continued from No. 7, page 115.]

In this, as in our first article, we intend merely to skim these Lectures, selecting certain opinions or points of practice only, so as to form a melange that may be interesting to the physician as well as the surgeon—to the physiologist, as well as to the general practitioner.

*Lecture* IV. This is on adhesive inflammation, and exhibits all the doctrines of Hunter, improved by modern observation and experiment.

It is well remarked by Sir Astley Cooper, that even in diseases we often see the wise provision of Nature—or, in other words, the wisdom and beneficence of the Creator. Thus, the serous membranes, as the pleura and peritoneum, are, perhaps, of all other parts, most prone to the adhesive inflammation; and well it is so, for were they equally disposed to the suppurative process, the shut cavities would be filled with purulent matter every time that inflammation occurred. In the severer forms, however, of pleuritis and peritonitis, this dangerous consequence of inflammation does actually take place, as pathology has too often proved. The mucous membranes, on the other hand, which have external openings, are little disposed to the adhesive, and very prone to the suppurative process—a circumstance wisely ordained, and for the most obvious reasons. Sometimes, however, where the mucous membrane, as of the urethra, is violently inflamed, the adhesive inflammation takes place.

"I witnessed the following curious example of this circumstance: a kangaroo was brought to me for dissection, from Exeter Change; its bed of straw had caught fire, and, although it was very soon extinguished, the animal died; and the proprietor, knowing that it had not been severely burnt, was at a loss to account for its death. Upon examination, the bladder was excessively distended with urine, and it was retained in consequence of the closure of the urethra by the adhesive inflammation; the penis having been injured by the fire, the inflammation that followed was violent, and, being adhesive, closed the urethra." 96.

The same observation applies to the air passages. In certain degrees of phlogosis there, mucus, pus, and adhesive matter are thrown out. We have seen an instance where the rima glottidis was hermetically sealed by adhesive inflammation. Bronchotomy saved life.

The uses of the adhesive inflammation in various operations, as well, as in diseases, are well known. Speaking of ligatures, Sir Astley recommends that one end only should be cut away. The practice of cutting both close to the knot has been abandoned. A cat-gut ligature applied by Sir Astley, to the femoral artery of an old man, was absorbed, but the experiment never again succeeded.

It is curious to observe how fashions in surgery change, even in the course of a very short time. Thus, it is only a few years —(nay it is now perhaps very generally done in the country and colonies) since it was the practice, in amputation, to apply numerous strips of adhesive plaster across the face of the stump —then lint—next plaster—and last of all compresses and roller, or a night-cap.

"If a surgeon were to do this now, he would be laughed out of the operating theatre; and very deservedly, because he would prevent the success of the adhesive process by undue heat in the limb.

"All that is necessary to do is, to use three strips of plaster over the wound, and one circular piece; if the weather be hot, to apply spirit of wine and water; and if cool, to keep the limb quiet. The object is, to prevent the inflammation passing beyond the adhesive stage; for then suppuration must be the result." 108.

We believe that the simple method now adopted is the best —but we are far from thinking that the plan pursued a few years ago, "prevented the success of the adhesive process," if we may judge by the comparative results of the two methods. Adhesion appears to us not a whit more frequent than it was twenty years ago. But great inconvenience, if not detriment, attended the complicated dressings of that period, and we are not sorry to see them laid aside.

Surgeons, in general, open wounds too soon—in amputations particularly. Sir Astley recommends one strip to be removed about the fourth day, to let out any matter which may have collected. "In six or eight days after the operation, it will be proper to dress the stump, and then to re-apply a strip of plaster before you remove the whole dressing." Where only three strips, however, are applied across the face of the stump, and where intervals are left for the escape of matter, there cannot always be a necessity for disturbing the stump at all on the fourth day. These observations apply, of course, to wounds as well as to amputations.

The public mind was agitated some time ago by the case of wounded knee-joint, which terminated fatally at St. George's Hospital, and where much blame was attempted to be thrown on the Surgeon for the treatment pursued. The following are Sir Astley's directions for the management of a wound of this kind.

"If, on the contrary, the surgeon brings the edges of the wound immediately together, and takes advantage of the adhesive inflammation to close the wound, the patient escapes from local or constitutional irritation, and in a fortnight is free from danger, and has scarcely suffered from the injury. He effects this object by bringing the edges of the wound together, by a fine suture, a plan to which some surgeons object; but when the wound is direct into the joint, it secures best the safety of the patient, as the secretion of synovia has a constant tendency to prevent adhesion, and to separate the plaster. Let the suture penetrate the skin only, avoiding the ligament; apply a piece of lint over it wetted in the patient's blood, and strips of adhesive plaster over the lint. A roller is to be gently bound round the knee, and to be kept constantly wet with the liq. plumb. acet. c. spir. vini; and a splint is to be placed behind the joint to preserve perfect rest." 111.

It would have been proper, however, to add that, in the event of the adhesive inflammation overstepping the salutary bounds, the most decisive steps are to be taken to check it, by numerous leeches, cold evaporating lotions, and constitutional remedies of the antiphlogistic class.

We must pass over entirely the subjects of suppuration and ulceration, on which the able author, and also his intelligent editor, have made numerous admirable observations. There is a remark on the subject of those chronic abscesses which not unfrequently form between the cranium and pericranium, in bad constitutions after long courses of mercury, which deserves notice. In such cases, Sir Astley recommends that the abscess should not be opened, unless there be a blush on the skin. Where the integuments are not discoloured, the matter will be absorbed, however distinct the fluctuation, by a proper exhibition of the decoction of sarsaparilla, assisted occasionally by small doses of the oxymurias hydrargyri. This we have repeatedly seen—and what is more, we have observed the imprudent opening of these pericranial abscesses, followed by very tedious and ill-conditioned sores and exfoliation of the bone, which might otherwise have been avoided.

The prevention of scars in the necks of females, where scrofulous abscesses are apt to form, has not escaped the attention of Sir Astley Cooper. In these cases, much depends on the constitutional treatment. Aperients, with calomel and rhubarb, should be administered—evaporating lotions applied—nutritious but not stimulating food given. The abscesses should be opened with a very small knife, and before the skin is much affected, as soon as a blush has appeared, and then the greatest care is to be taken that all the matter is pressed out, in which case the parietes of the abscess will often heal by the adhesive inflammation, and scars will be prevented. Sir Astley directs the opening in these abscesses to be made transversely, and not in the direction of the axis of the neck, in order that the seam may be concealed among the creases or folds of the skin. They should not be opened, however, "when they have a purple blush upon them, like the hue of a grape." The skin is thin, and will then slough.

Sir Astley believes, with most physiologists of the present day, that hectic fever is not to be attributed to the absorption of pus—but is to be looked upon as "merely the result of the efforts of the constitution to repair an injury, or to cure a disease." With this explanation, we believe we must rest satisfied at present. We have no other for any of the reactions which take place in the constitution, when any morbid cause is applied—as the contagion of small-pox, the marsh miasma of a fen, or a musket shot through the thigh.

Sir Astley makes a concluding remark on the influence which the admission of air into cavities is supposed to have in producing local irritation. The constitutional disturbance takes place about the third day, but it is evidently not attributable to the admission of air, for this fluid may be thrown into cavities and into the cellular membrane, without any such consequences ensuing.

"If a wound be made into any cavity of the body, be it an abscess or a natural cavity, soon after the vessels of the part are divided, inflammation arises to heal the wound, whether it be exposed to the air or not. If it heal by adhesion, the influence is slight and directly terminates; but if the adhesive inflammation be insufficient or imperfect, then a suppurative inflammation follows, and granulations arise, which process produces violent influence both upon the part and constitution. The cause is, therefore, the division of the blood vessels, and not the presence of air; and its degree depends upon the ease or difficulty with which the injury is repaired." 159.

The lecture on granulation contains many interesting remarks. The mode in which these are formed, and their physiological properties are minutely considered by our able author. He looks upon a granulation, and consequently on the surface of an ulcer, as a gland, where a secretion is formed by the arteries, and the remaining blood is returned by the veins. These granulations are not good absorbing surfaces in ulcers recently formed; but, if the ulcers have existed for some time, the absorbent vessels (including the veins of course) readily take up substances applied to them. In this way persons are often salivated from injections of oxymuriate of mercury into sinuses.

"I have known what is commonly called the black lotion, which is composed of the liquor calcis, and the submuriate of mercury, when applied to the surface of ulcers, produce an effect upon the mouth in persons who are easily affected by mercury. I believe that the lotion of the liquor calcis and calomel often produces good effects in sores, by the mercurial action which it excites in the system, and not merely by its local effects on the sore to which it is applied." 164.

The bad effects resulting from the application of poisons, such as arsenic, to sores, are well known. Granulations have nerves as well as blood-vessels, but some are almost insensible, as those which spring from bone. Others are highly sensitive.

When on the subject of cicatrization, Sir Astley observes that all parts of the body are capable of reproduction except two—muscle and cartilage. When the *former* is divided it unites by a tendinous substance, and the *latter*, by an ossific union. Cuticle and rete mucosum are reproduced, but the latter slowly. The new skin of a wounded negro is at first red, but, in time, the rete mucosum becomes as black as in any other part of the body.

The Lecture on Ulcers, as may be supposed, contains important practical matter, from the author's wide experience. In gangrenous ulcers Sir Astley gives the preference to nitric acid, as an external application—fifty drops to a quart of distilled water—increasing the acid occasionally. Oiled silk should be applied to the wound to prevent evaporation and preserve the moisture of the linen. Sir Astley also recommends a solution of nitre—one drachm to the pint of water. As an internal medicine in sloughing and gangrenous sores, Sir A. gives 20 drops of laudanum thrice a day, with ten grains of carbonate of ammonia, in camphor, julep, and some tincture of cardamoms. Port wine must be allowed—and even spirits to those who have been accustomed to them.

To irritable ulcers, which are very troublesome to the practitioner, Sir Astley recommends an ointment composed of spermaceti ointment, unguentum hydrargyri, and powdered opium, applied thrice a day. Mr. Tyrrell, however, prefers a lotion. We shall give his own words.

"In these cases I usually employ a lotion, composed of lime water, mucilage, and opium, in preference to the ointment, for reasons I have before mentioned. As a general remedy to irritable ulcers, I can with confidence recommend it strongly, as I have had ample opportunity of witnessing its good effects. It is applied on lint or soft linen to the ulcerated surface; and a portion of oil silk, or a light poultice, is placed over it, to prevent the lint from drying. In preparing the lotion, the opium must be dissolved in the lime water, and the solution is then to be filtered, to get rid of all extraneous particles, after which the mucilage is added: the proportions are as follow:

"℞ Liquor. calcis ℔j.

Extract. opii ʒj.

Mucilag. acaciæ ℥ij. M. fiat lot." 194.

"The internal remedies you ought to exhibit," says Sir Astley, "are calomel and opium—a grain and a half of calomel, and a grain of opium morning and evening."

"It should not be carried so far as to produce ptyalism, or to affect the constitution severely; but it should be given so as to restore the secretions, and to diminish the excitement of the nervous system. The calomel will do the first, and the opium will lessen the nervous irritability. The treatment of inflammation has been improved of late, by exhibiting calomel and opium. The effect of this medicine in inflammation may be seen in the disease called iritis. Here calomel and opium must be exhibited: nor should a deposit of adhesive matter into the anterior chamber of the eye, be any bar to their use. Give five grains of calomel and a grain of opium night and morning; and in the space of a week, if the eye has not suffered so much as to be disorganized, this remedy will correct the inflammation, and vision will be restored." 195.

*Phlebectasia.* Our readers are aware that we laid before them, in our last number, a paper on the subject of varicose veins, from the French; in which it would appear that the practice of ligature has been more successful on the Continent than in this country. Sir Astley sets his face against the practice entirely, and seems to trust to the ancient Hippocratic treatment of puncturing the varicose veins frequently—rest—and proper bandaging. Our author avers, that he had rather have the femoral artery than the saphena vein tied in his own person.

In the lecture on gangrene, Sir Astley Cooper evinces a rare combination of physiological knowledge, philosophical induction, and minute practical observation. The causes of death, in a part, are properly ascribed to too great action, after exposure to cold or other morbific agents,—and to a defect of vital power, without any preceding inflammation, as in the toes of old people, and where the circulation of the blood is prevented by mechanical means. The various steps which Nature takes in separating the dead from the living parts, are faithfully and admirably described—and the best treatment is clearly laid down. Soothing applications are necessary to a part which is falling into gangrene—blood-letting, to any extent, is dangerous, especially in this metropolis, notwithstanding that excessive action is going on in the vessels of the part. Some calomel and opium should be given at night, to restore the secretions of the liver and intestinal canal, and quiet irritability. Stimulation must be cautiously used in the beginning, and gradually increased when gangrene has actually taken place. Opium, ammonia, and the sulphate of quinine, are the internal remedies recommended by our intelligent author; but we were rather surprised to find him omit the mention of wine, which, in such cases, combines the stimulating properties of ammonia with the tranquillizing ones of opium.

It is well known that Nature will often be able to separate a whole limb, bone and all, that has fallen into sphacelus. The process is somewhat more tedious, however, than amputation by a modern surgeon, who performs the operation while a few grains of sand are running through a minute glass. The propriety of amputating in gangrene is discussed by Sir Astley, who comes to the conclusion, that we are not to use the knife, "till the sloughing process has commenced, and healthy granulations are to be seen on the sore." This principle we believe to be the correct one, although it has been questioned, and contravened in practice by some eminent Continental surgeons, especially the Baron Larrey. He performed some amputations, and with success, while the gangrene was spreading, and before any line of demarcation was established between the dead and living parts.—But a few instances of success do not affect the general principle, though, we think, that particular circumstances may authorise us to deviate from the precept laid down by our present author, and, indeed, by the general voice of the profession.

The dry or white gangrene of old persons is not satisfactorily accounted for—at least by the common explanation given—ossification of the arteries. It has not taken place where the arterial system was very generally ossified, and it has often taken place where there was no ossification of the vessels. We look upon it, therefore, as a coincidence rather than a consequence. We have seen several instances of mortification of the lower extremities, in young persons, from enlargement of the heart—and we suspect that disease of the central organ of the circulation is more frequently the cause of the dry gangrene of old people, than disease of the arterial conduits leading therefrom. Poultices of oatmeal and port wine are recommended; with opium and ammonia internally; but the disease is generally fatal in the end, though not always so. Sir Astley has known a single toe—all of them—and a portion of the foot, slough away, and yet the patient recover.

*Erysipelas.* Few diseases have excited more discussion, and produced more discrepancy of opinion, in respect to treatment, than this. But the fact is, that in different constitutions —and especially in different atmospheres, as of town and country—the treatment must vary materially. We fully agree with Sir Astley, that in this metropolis, whose *atmosphere* contains so small a proportion of *air*, the following plan will be found the best, as a general rule.—"At first give calomel, for the purpose of restoring the secretions of the liver and intestines, and the liquor ammoniæ acetatis with antimony, to act upon the secretion of the skin ;—and then give the sulphate of quinine. It is a most powerful tonic, excites in the stomach a genial warmth, and often will remain in that organ when bark will be rejected." This we believe. But, alas ! the sulphate of quinine is too good (or rather too *dear*) a medicine to remain long unadulterated. We have the best means of knowing that this important preparation is now generally sophisticated—and with what ? with *Epsom salts !* But more of this on a future occasion. We shall here introduce a case as stated in a note by Mr. Tyrrell, illustrative of the good effects of this much abused medicine.

"I take the liberty of introducing the following case of erysipelas, as remarkable on account of the great extent of the disease, and also as showing the beneficial effects produced by the exhibition of sulphate of quinine.—July 5th, 1824, J. Hawks, æt. 46, a plasterer by trade, was admitted into St. Thomas's Hospital, on account of an erysipelatous inflammation of the right leg, resulting from a contusion near the internal malleolus. The erysipelas appeared on the evening of the same day on which he received the injury, and he had been previously in an indifferent state of health. When admitted, the inflammation extended nearly to the groin, and completely surrounded the extremity ; his bowels were confined, and he complained of heat and pricking pain in the affected limb : the constitutional irritation was also very considerable. He was ordered to take fifteen grs. of the compound pill of colocynth with calomel, and to apply a spirit wash over the inflamed surface: also, to allay irritability, small doses of calomel and opium were directed to be given every night. On the following day, my colleague, Dr. Elliotson, saw the patient with me, and ordered, in addition to the calomel and opium, five grs. of the sulphate of quinine, five drops of dilute sulphuric acid, and two oz. of water, to be taken every six hours: with an allowance of three pints of milk daily. On the 7th, the inflammation had extended a little, and vesicles had formed on the thigh :

the former medicines were continued, and castor oil ordered to be taken occasionally as required. 9th, As the pulse was rather feeble, I desired he should have a pint of porter daily, in addition to the other remedies. On the 10th, the erysipelas had extended up the side nearly to the axilla, on the abdomen almost to the median line; it also covered the whole of the nates and back: a spot on the dorsum of the foot appeared disposed to become gangrenous, and he was altogether weaker. He was ordered to take the quinine every four hours, to have beef steaks daily, with another pint of porter, to continue the calomel and opium, and also the spirit wash, excepting to the foot, which was to be poulticed. 11th. The calomel omitted, as his mouth became slightly affected. 12th. To continue and have four ounces of sherry in addition. 13th. Another pint of porter was ordered. From this period he continued to improve, and he took daily beef steaks, three pints of porter, four ounces of sherry, also the sulphate of quinine, every four hours, and the opium at night; the slough on the foot separated quickly from the application of the nitric acid lotion, and the erysipelas gradually disappeared, causing a loss of the cuticle. Matter formed in the cellular texture on the calf of the leg and at the under part of the thigh; it was discharged by incision, and the parts quickly healed.

"At one time the erysipelas extended from the toes of the right lower extremity, to the occiput, occupying the whole of the limb, the back and nates, and reaching as far on the abdomen of the same side as the linea alba.

"I attribute the favourable termination of this case, in great measure, to the judicious employment of the sulphate of quinine, by my colleague, Dr. Elliotson, which appeared to give tone and vigour to the system, as well as to allay the nervous irritability, so constantly attending this form of inflammation." 248.

Among the lower classes, who are in the habit of drinking gin and porter in abundance, we must give some of the same stimulus, even while labouring under disease, and especially erysipelas. The truth of this was proved some 60 years ago, in a paper by one of the Monros. Sir Astley recommends, as an application, the camphorated spirits of wine, in the first stage; and, when the vesications break, the old remedy, powdered starch. If gangrene supervene, the wine poultice, or the nitrous acid lotion. In the erysipelas phlegmonoides, the incisions, first recommended by Mr. A. C. Hutchison, are to be put in force.

The three concluding lectures of the first volume, are on injuries of the head—viz. concussion, compression, fractures of the cranium, and wounds of the brain. In the first volume of this series, p. 464 *et seq.* we have given a summary of our author's opinions and practice in these important accidents, and therefore, we need not resume the subject in this place. In our next number we shall take up the second volume of these interesting lectures, and direct our attention more especially to the medical surgery of the work, collating it with other sources of information, foreign and domestic.

